# Phytochemical Analysis and Antioxidant and Antidiabetic Activities of Extracts from *Bergenia ciliata*, *Mimosa pudica*, and *Phyllanthus emblica*

**DOI:** 10.1155/2022/4929824

**Published:** 2022-07-07

**Authors:** Basanta Kumar Sapkota, Karan Khadayat, Kabita Sharma, Bimal Kumar Raut, Dipa Aryal, Bijaya Bahadur Thapa, Niranjan Parajuli

**Affiliations:** Biological Chemistry Lab, Central Department of Chemistry, Tribhuvan University, Kirtipur, Kathmandu 44618, Nepal

## Abstract

Diabetes is a metabolic disorder of high blood sugar levels which leads to various chronic health-related complications. The digestive enzymes *α*-amylase and *α*-glucosidase play a major role in the hydrolysis of starch to glucose; hence, inhibiting these enzymes is considered an important strategy for the treatment of diabetes. Medicinal plants such as *Bergenia ciliata*, *Mimosa pudica*, and *Phyllanthus emblica* are commonly used in traditional remedies due to their numerous health benefits. This study aimed to determine the phytochemicals as well as TPC and TFC contents in these plant extracts along with their antioxidant and enzyme inhibitory activity against *α*-glucosidase and *α*-amylase. The ethyl acetate extracts of selected plants have shown higher TPC and TFC contents. The aqueous extract of *B. ciliata* (IC_50_: 16.99 ± 2.56 *μ*g/mL) and ethyl acetate extract of *P. emblica* (IC_50_: 11.98 ± 0.36 *μ*g/mL) and *M. pudica* (IC_50_: 21.39 ± 3.76 *μ*g/mL) showed effective antioxidant activities. Furthermore, ethyl acetate extract of *B. ciliata* showed significant inhibitory activity against *α*-amylase and *α*-glucosidase with IC_50_ values of 38.50 ± 1.32 *μ*g/mL and 3.41 ± 0.04 *μ*g/mL, respectively. Thus, secondary metabolites of these medicinal plants can be repurposed as effective inhibitors of digestive enzymes.

## 1. Introduction

Diabetes, a global health problem, is a chronic metabolic disorder due to dysfunction in the production and/or utilization of insulin [1]. Diabetes has become a subject of concern worldwide due to its chronic health complications such as cardiovascular diseases, nephropathy, neuropathy, lower-limb amputations, retinopathy, and others, leading to complicated lifestyle and mortality [2]. There is an increased risk of infection in diabetic patients due to the disruption in the immune system. Secondary metabolites in natural products are the source of major lead compounds for the optimization of pharmacological activities due to their less toxicity and moderate side effects [3]. The metabolites such as flavonoids, alkaloids, and polyphenols have a wide range of biological activities such as antioxidant, antimicrobial, anti-inflammatory, anticancer, antidiabetic, and other activities [4]. Besides, polyphenols can reduce oxidative stress and can inhibit enzymes of carbohydrate digestion and play a significant role in preventing hyperglycemia [5, 6].

*B. ciliata* (family Saxifragaceae), known as Pakhanbhed in Nepali, is traditionally used for the treatment of diabetes in local communities [7]. Besides that, crude extracts of rhizomes and leaves of this species were studied for anti-inflammatory, antimicrobial, anticancer, antidiabetic, antioxidant, and others activities [8–12], and its major phytoconstituents include bergenin, gallic acid, gallicin, tannic acid, catechin, (-)-3-0-galloylepicatechin, (-)-3-0-galloylcatechin, stigmasterol, B-sitosterol, galloylated leucoanthocyanidin-4-glucoside, allantoin, and afzelechin [13]. *P. emblica*, commonly known as Amla of Euphorbiaceae family, possesses several pharmacological properties such as antioxidant, antimicrobial, antifungal, anticancer, antidiabetic, and other properties [14]. Some of the major chemical constituents of this species are chebulagic acid, chebulic acid, corilagin, phyllanemblinin A, gallic acid, ellagic acid, malic acid, mucic acid, (-)epicatechin, and mallonin [15]. *M. pudica* of the family Fabaceae commonly found in South Asian countries possesses antidiabetic, antibacterial, wound-healing, antivenom, and anticancer properties and is traditionally used for treatment of fever and dyspepsia [16]. Phytochemical analysis revealed the presence of flavonoids C-glycosides, sterols, terpenoids, tannins, fatty acids, p-coumaric acid, mimopudine, and mimosine in *M. pudica* [17]. This study aims to evaluate antidiabetic activity based on *α*-amylase and *α*-glucosidase as well as antioxidant activities of three medicinal plants found in Nepal.

## 2. Material and Methods

### 2.1. Chemicals

Methanol, ethanol, ethyl acetate, dichloromethane, and hexane were purchased from Thermo Fischer Scientific (India). Gallic acid and quercetin were purchased from HiMedia (India). *α*-Glucosidase (*Saccharomyces cerevisiae*), 4-nitrophenyl-*α*-D-glucopyranoside (pNPG), *α*-amylase from porcine pancreases, 2-chloro-4-nitrophenyl-*α*-D-maltotrioside (CNPG3), acarbose, and neomycin were obtained from Sigma-Aldrich (Germany).

### 2.2. Medicinal Plant Extracts

*B. ciliata*, *M. pudica, and P. emblica* were collected from the different geographical regions in Nepal and were botanically identified by the National Herbarium and Plant Laboratories (NHPL)/KATH, Godawari-3, Lalitpur, and their voucher specimen were assigned (Table 1). The plant materials were shade-dried at room temperature and then pulverized by the mixture and soaked into methanol for 24 hrs following the cold percolation protocol. Then they were filtered through Whatman Filter Paper 1 and collected in a conical flask, and the same process was repeated thrice. The collected methanol was evaporated from primary extracts using a rotary evaporator under reduced pressure at 40°C. Then, the percentage yield was calculated using the dry weight of extract and the dry weight of powder soaked in methanol. The secondary extracts were prepared by dissolving the primary extract in water and then fractionated with different solvents such as hexane, dichloromethane, and ethyl acetate based on increased polarity [18]. The percentage yield was calculated by the given formula:

(1) $$\begin{array}{c} \%\text{Yield}=\frac{\text{Dry weight of extract}}{\text{Dry weight of a plant}}\times 100. \end{array}$$

### 2.3. Phytochemical Identification

The chemical method was used to identify phytochemicals present in the extracts. Different tests such as steroids, alkaloids, phenols, terpenoids, tannins, and glycosides were performed as previously described methods [19–21]

### 2.4. Determination of Total Phenolic and Flavonoid Contents

The total phenolic and flavonoid contents (TPC and TFC) were determined using the method described earlier. For the determination of TPC, Folin-Ciocalteau's, and for the determination of TFC, the aluminum trichloride method was used [22–24]. Both assays were performed in 200 *μ*L, and absorbance was taken using a microplate reader (Synergy LX, BioTek, Instruments, Inc., USA). Gallic acid and quercetin were used as the standard to generate calibration curves at various concentrations and expressed as gallic acid and quercetin equivalent (mg GAE/g and mg QE/g), respectively.

### 2.5. Antioxidant Assay

The antioxidant activity of different extracts was evaluated against DPPH radicals according to the method described earlier [25, 26]. Exactly an equal volume of samples with different concentrations was mixed with 0.1 mM DPPH reagent to maintain a final volume of 200 *μ*L. Then, it was incubated in dark at room temperature for 30 min, and then absorbance was recorded at 517 nm. Quercetin was used as the standard to compare the antioxidant efficacy of plant extracts. The given formula determined the percentage scavenging:

(2) $$\begin{array}{c} \text{Scavenging DPPH radical}=\frac{{\text{ OD}}_{\text{control}}-{\text{OD}}_{\text{test sample}}}{{\text{OD}}_{\text{control}}}\times 100. \end{array}$$

### 2.6. *In Vitro α*-Glucosidase Inhibition Assay

The *α*-glucosidase inhibition was determined following the method described earlier. The test samples in 30% DMSO were mixed with enzyme (0.2 units/mL) in 100 mM phosphate-buffered saline (pH 6.8) and then preincubated at 37°C for 10 min. The reaction was started by adding pNPG as substrate (0.7 mM) and incubated for 15 min at the same temperature [27]. The absorbance was measured at 405 nm using a microplate reader and inhibitory activity was calculated using the following formula:

(3) $$\begin{array}{c} \text{\% Inhibition}=\frac{{\text{ OD}}_{\text{control}}-{\text{OD}}_{\text{test sample}}}{{\text{OD}}_{\text{control}}}\times 100. \end{array}$$

### *2.7*. *In Vitro α*-Amylase Inhibition Assay

The *α*-amylase inhibition was determined using a literature method. The test samples in 30% DMSO were mixed with enzyme (1.5 units/mL) in 50 mM phosphate-buffered saline (pH 7.0, 0.9% NaCl) and then preincubated at 37°C for 10 min. The reaction was started by adding CNPG3 as substrate (0.5 mM) and left for 15 min for incubation at the same temperature [28]. The absorbance was measured at a 405 nm microplate reader and inhibitory activity was calculated using the given formula mentioned earlier (equation (1)).

### 2.8. Statistical Analysis

The Gen5 Microplate Data Collection and Analysis software was used for result processing, followed by Microsoft Excel. The data were expressed as mean ± standard error of the mean. The IC_50_ values were determined using GraphPad Prism (Version 8) software. The XLSTAT software (Addinsoft, USA, NY) was used to perform principal component analysis and correlation analysis.

## 3. Results

### 3.1. Percentage Yield and Phytochemical Identification

The percentage yield of selected plants was calculated with highest being obtained in *P. emblica* (39.5%) followed by *M. pudica* (17.6%) and *B. ciliata* (15.86%). Our study indicated that the highest yield is obtained from fruit, whole plant, and stem, respectively. The phytochemical identification indicated the presence of alkaloids, phenols, flavonoids, terpenoids, tannins, and glycosides as shown in Table 2.

### 3.2. Analysis of Total Phenolic and Flavonoid Contents

The TPC and TFC of methanolic primary extracts and their partitioned fractions (secondary extracts) are mentioned in Table 3. Among all fractions, the EA fraction had the highest TPC, while, in TFC, crude and EA fractions had the highest contents. The TPC and TFC were expressed as mg GAE/g and mg QE/g of extracts using calibration curves of gallic acid and quercetin, respectively.

### 3.3. Antioxidant Assay

The antioxidant activities of different fractions were determined using the DPPH assay. The IC_50_ value of various solvent fractions showed medium-to-strong DPPH scavenging activity ranging from 11.98 to 141.53 *μ*g/mL. The potent activity was shown by ethyl acetate, as well as crude and aqueous fractions. The quercetin was used as standard and the IC_50_ value was found to be 2.86 ± 0.51 *μ*g/mL. The details about the IC_50_ values of each fraction are shown in Table 4.

### 3.4. Inhibition of Digestive Enzymes

The results revealed that the IC_50_ value of various solvent fractions showed medium-to-strong inhibitory activity. However, the ethyl acetate fraction showed strong activity against *α*-amylase, while, in the case of *α*-glucosidase, ethyl acetate, crude, and aqueous fractions showed significant activities. For both enzymes, acarbose was used as a positive control with IC_50_ values of 3.13 ± 0.14 *μ*g/mL and 2.06 ± 0.07 mg/mL for *α*-amylase and *α*-glucosidase, respectively. The details are given in Table 5.

### 3.5. Correlation Analysis between TPC, TFC, DPPH, *α*-Amylase, and *α*-Glucosidase

The Pearson correlation analysis was performed between TPC, TFC, DPPH, *α*-amylase, and *α*-glucosidase as shown in Table 6.

### 3.6. Principal Component Analysis

**Figure 1:**
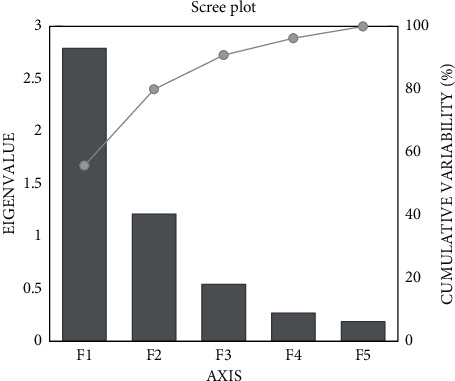
Scree plot showing eigenvalue and cumulative variability of studied parameters.

Principal component analysis (PCA) was performed on five different variabilities among varied solvent fractions to obtain information about the interrelationship among variables. Among the five components, PC1 and PC2 showed eigenvalues >1, while the remaining principal components had eigenvalues <1 and so have not been discussed further (Figure 1).

**Figure 2:**
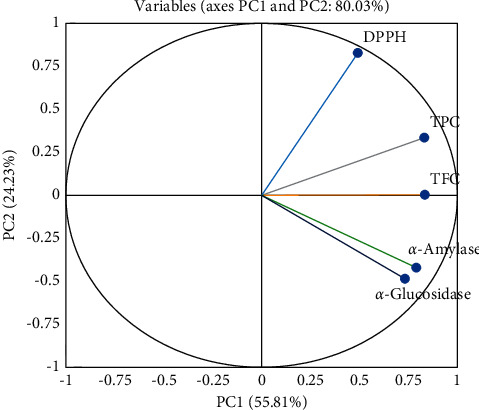
Loading plot of phytochemical content and pharmacological parameters.

The principal axis 1 (PC1) accounted for 55.807% of the variance, whereas principal axis 2 (PC2) accounted for 24.226% and they altogether accounted for 80.033% of the total variance of the data matrix, with PC1 being the prominent one (Figure 2).

The eigenvalues of two principal component axes among five were found to be more than one with 2.79 and 1.21, respectively. The principal component score plot produced from PC1 revealed that all variables were positively associated and, hence, showed a good correlation between them. PC1 showed positive factor loadings for all variables. TFC showed the highest factor loading at 0.834, followed by TPC with a 0.831 factor loading value depicting that TFC could be the best individual factor loadings selection. PC2 confirmed positive factor loadings for three variables, that is, TPC, TFC, and DPPH, while DPPH could be the best selection for individual factor loadings with a maximum value of 0.828 followed by TPC (Table 7). Figure 2 revealed the loading plot of phytochemical contents and pharmacological parameters. TPC, TFC, *α*-amylase, and *α*-glucosidase were found to have significant effects on PC1, while DPPH had strong influences on PC2.

## 4. Discussion

Diabetes patients are treated by controlling the blood glucose to a normal level, in both the fasting and postprandial states. *α*-Amylase is responsible for the hydrolysis of a 1,4-glucosidic linkage of starch, glycogen, and oligosaccharides. Then, *α*-glucosidase found on the brush border interface membrane of intestinal cells further breaks down the disaccharides into glucose, readily available for intestinal absorptions. One of the strategies to control diabetes is to inhibit these two enzymes and reduce the glucose absorption resulting from the breakdown of starch by these enzymes [29]. Therefore, an effective and nontoxic inhibitor of digestive enzymes from medicinal plants has been investigated for a long time [30].

This study is focused on the investigation of the antidiabetic potential of Nepalese medicinal plants, namely, *B. ciliata*, *M. pudica*, and *P. emblica*, which have been used in the formulation of traditional medicine for the treatment of several diseases [7, 8, 12, 14]. Here, phenolic and flavonoid contents of each solvent fraction of individual plants under study were determined. Our findings revealed that among methanolic extracts and their different fraction such as hexane, DCM, EA, and water-based on polarity, methanolic extracts, and EA fractions had shown the highest phenolic and flavonoid contents. Both phenolics and flavonoids play an important role in the antioxidant activity of their redox properties, acting as a reducing agent, as well as donors of hydrogen atoms [31]. Terpenoids are considered primary antioxidants as these compounds can donate hydrogen atoms to radicals, ultimately slowing down the lipid oxidation process [32]. Phytochemical identification revealed the presence of alkaloids, tannins, terpenoids, glycosides, steroids, saponins, and anthocyanin that might inherent the antioxidant activity. For the solvent fraction, ethyl acetate showed strong antioxidant activity with IC_50_ values of 28.15 ± 0.17 *μ*g/mL, 46.06 *μ*g/mL, and 11.98 ± 0.36 *μ*g/mL for *B. ciliata, M. pudica,* and *P. emblica*, respectively, followed by medium scavenging activity of methanol and aqueous fractions, which are comparable to the finding of previous reports [33–36]. Similarly, our result resembles the study that showed significant *α*-glucosidase activity in ethyl acetate, methanol, and aqueous fraction, with strong activity against *α*-amylase enzyme in ethyl acetate fractions [7, 36–38].

A previous study on *B. ciliata* showed TPC of 473.4 ± 15.1 mg GAE/g and TFC of 89.9 ± 0.1 mg QE/g from methanol and TPC of 249.7 ± 1.3 mg GAE/g and TFC of 208.4 ± 0.6 mg QE/g from ethyl acetate, while in our study the TPC and TFC were reported as 155.83 ± 1.51 mg GAE/g and 47.26 ± 1.21 mg QE/g from methanol and 168.24 ± 1.17 mg GAE/g and 28.49 ± 0.67 mg QE/g from EA fraction. The antioxidant was reported as 53.5 *μ*g/mL from methanolic leaf extract, 2593.3 *μ*g/mL from ethyl acetate, and 3026.7 *μ*g/mL from hexane extract [39], while in our study we found 52.6 ± 3.63 *μ*g/mL from methanol, 18.42 ± 1.29 *μ*g/mL from EA, and 60.24 ± 2.19 *μ*g/mL from hexane. A previous study had shown 84.3 ± 13.2% inhibition in EA fraction and 65.3 ± 2.7% inhibition in water fraction partitioned from the methanolic extract. Two phenolic compounds, namely (-)-3-O-galloylcatechin and (-)-3-O-galloylepicatechin, were isolated from ethyl acetate fraction of *B. ciliate* [7]. Both compounds, largely present in tea, act as a strong rat intestinal *α*-glucosidase and porcine pancreatic *α*-amylase inhibitor via prohibition of the progressive deterioration of pancreatic beta-cell function as a result of oxidative stress [40].

*M. pudica* was reported to have TPC values of 28.523 ± 5.296, 57.431 ± 1.096, and 42.550 ± 2.228 mg GAE/g, respectively, for hexane, methanol, and ethyl acetate extract as compared to 41.45 ± 2.6, 131.78 ± 1.53, and 164.21 ± 1.81 mg GAE/g of our study. Likewise, TFC value was found to be 0.927 ± 0.461, 16.97 ± 1.472, and 3.90 ± 0.059 mg QE/g, respectively, whereas our results showed flavonoid content of 11.77 ± 1.18, 20.11 ± 0.75, and 64.89 ± 4.70 mg QE/g. In accordance with IC_50_ values (81.28 ± 8.23 *μ*g/mL for hexane, 34.35 ± 5.11 *μ*g/mL for methanol, and 21.39 ± 3.76 *μ*g/mL for ethyl acetate) of our research, antioxidant activities for these extracts were also found to be 92.302 ± 0.0077, 7.18 ± 0.0005, and 49.59 ± 0.0024 *μ*g/mL, respectively [37]. The study revealed that ethyl acetate extract exhibits better DPPH scavenging activity with a minimum IC_50_ value of 46.06 *μ*g/mL and higher TPC and TFC values of 15.64 ± 1.31 mg of GAE/100 g and 1.97 ± 0.47 mg of QUE/100 g, respectively, which are following our study. The synergistic effect of bioactive components like flavonoids and phenolic components found in them may be responsible for the antioxidant and biological activity of further inhibiting the progression of oxidative stress-induced disorders [35]. Stigmasterol operates as a metal chelator, peroxide, and lipid peroxide scavenger due to the unsaturation of the rings, which attributes to conjugation [41]. The mechanism of action of quercetin, an antioxidant compound, is due to the combined impact of possessing 3′,4′-dihroxy group coupled with 5′–OH and 3″ substitution, while that of avicularin may be due to sugar moiety linked to the quercetin structure leading to a considerable reduction in molecule's scavenging ability [42, 43]. Similarly, stigmasterol, quercetin, and avicularin isolated from ethyl acetate fraction of *M. pudica* showed *α*-glucosidase activity with an IC_50_ value of 91.08 ± 1.54, 75.16 ± 0.92, and 481.7 ± 0.703 *μ*g/mL, respectively [44].

The study done on *P. emblica* showed 439.9 ± 1.3 mg/g TPC and 12.6 ± 0.2 *μ*g/mL IC_50_ values for antioxidant wandhile 62.5 ± 0.7 mg/g TPC and 142.6 ± 5.3 *μ*g/mL IC_50_ values for antioxidant from an aqueous fraction [45]. In our study, 172.26 ± 3.61 mg GAE/g and 154.62 ± 3.29 mg GAE/g TPC were found from EA and water fraction. The IC_50_ values of 11.98 ± 0.36 and 22.34 ± 2.71 *μ*g/mL antioxidant were reported from EA and water fraction. Likewise, IC_50_ values of 5.68% *w*/*v* (56.8 mg/mL) and 0.87% *w*/*v* (8.7 mg/mL) were reported for *α*-amylase and *α*-glucosidase in aqueous fractions [36] as compared to 8.22% inhibition at 500 *μ*g/mL and IC_50_ value of 70.52 ± 3.65 *μ*g/mL in our study, respectively. Studies have shown that the major constituents like gallic acid, ellagic acid, and quercetin, along with other natural compounds, are responsible for a strong antioxidant effect as well as an antidiabetic effect [46–48]. Antioxidative stress activity of these compounds is due to their capability to inhibit the release of malondialdehyde (MDA) from RIN cells along with the reduction in the level of nitric oxide (NO) and glutathione (GSH) that are responsible for mitigating inflammatory responses [49, 50]. Gallic acid on improving the translocation and activation of GLUT4 in 3T3-L1 adipocytes [51] and PI3K/p-Akt-dependent pathway [52] exhibits its blood glucose-lowering activity. Besides that, gallic acid also prevents the apoptosis of pancreatic *β*-cells and acts as an insulin secretagogue [53]. Ellagic acid has antidiabetic properties due to its effect on pancreatic *β*-cells, which promote insulin production and reduce glucose intolerance [54]. Likewise, quercetin, one of the major constituents found in fruits of this species, is considered a potential antidiabetic drug due to its action via the combined effect of PPAR-*γ* with glycogen phosphorylase [55]. Similarly, compounds like chebulagic acid and corilagin act as a *α*-glucosidase inhibitor by inhibiting the glucose absorption [56, 57]. Previously, *in vitro* and *in vivo* study had shown the role of condensed and hydrolyzed tannins to control postprandial blood sugar levels in diabetes via inhibiting salivary and pancreatic *α*-amylases along with intestinal absorption of starch [58].

Correlation, a statistical analysis, is used to measure the relationship between different variables, with changes in one variable associated with changes in another, in either the same direction (positive correlation) or the opposite direction (negative correlation) [59]. In this study, significant positive correlations were observed between TPC with TFC, DPPH, and *α*-glucosidase inhibition. However, TFC and *α*-glucosidase inhibition have a significant correlation with *α*-amylase inhibition. The phenolic content and antioxidant activity were found to have a significant linear correlation, indicating that phenolic compounds could be responsible for antioxidant activity [35]. An increase in oxidative stress can cause insulin resistance, impaired insulin secretion, and late diabetic complication. Antioxidants, by inhibiting lipid peroxidation, can play an important role in the management of type 1 and type 2 diabetes mellitus [60]. Studies showed that there is a positive correlation between TPC, TFC, and antioxidant activities [61]. As a result, our findings can be correlated to the prior study demonstrating the positive role of phenolic and flavonoid content in free radical scavenging activity [62]. The positive correlation between TPC, TFC, *α*-amylase inhibition, and *α*-glucosidase inhibition is also consistent with a previous study which exhibited that TPC, TFC, and digestive enzyme inhibitory activities are positively correlated demonstrating TPC and TFC as the contributors to the inhibition of digestive enzymes [63, 64].

Principal component analysis (PCA) is a multivariate statistical technique used for analyzing the description of large datasets and retrieving the most useful statistics [65]. The PCA was carried out on all variables simultaneously to divide the pattern of variation. In this study, 2 principal components accounted for 80.03% of total variation with 55.81% and 24.23%, respectively. The correlation between original variables and the factors derived from PCA is called factor loading, which ranges from −1 to 1, where the value of −1 or 1 represents a strong correlation between both, while a value close to 0 represents a weak correlation. An absolute value of more than 0.4 represents 16% total variation and should only be interpreted according to Field (2005). All the variables had positive factor loading in PC1 and one variable from the recognized parameter was chosen based on individual loading [66].

**Figure 3:**
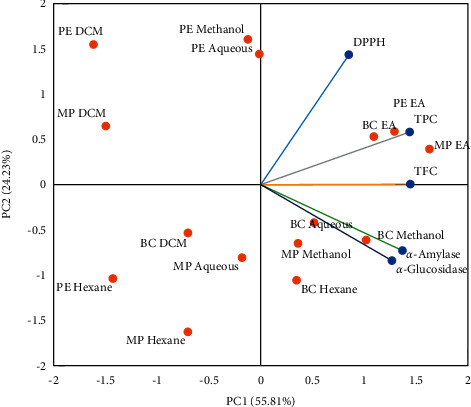
Factor score of 15 fractions of 3 medicinal plants in PC1 (*x*) and PC2 (*y*).

The factor score can be used for multivariate classification of different fractions of three mentioned plant species by plotting in the two dimensions with PC1 scores (*x*-axis) against PC2 scores (*y*-axis). The classification based on factor score is shown in Figure 3. The different fraction by variable biplot effectively revealed the visual comparison among all fractions based on multiple variables and also showed interrelationships among the variables. The angles between the vectors and the distance of the fractions from the origin of the biplot were used to extract important information. If the angle between two variables vectors is <90°, then the correlation between the traits is positive; if the angle is >90°, then variables show a negative correlation, while if the angle is equivalent to 90°, then variables show no dependency on each other [67]. The angle between two variables, DPPH and *α*-glucosidase, was >90°, so there is a negative correlation between them, while the remaining variables have <90° angle and showed a positive correlation. In our study, we observed ethyl acetate, methanol, and aqueous extracts are mainly responsible for their activity against different variables.

## 5. Conclusions

In conclusion, the study found that the ethyl acetate fraction has greater TPC and TFC content along with the potent antioxidant activity. The inhibitory activity of the ethyl acetate fraction against *α*-amylase was higher than that of the aqueous fraction against *α*-glucosidase. Thus, the ethyl acetate fraction had significant activity as compared to other fractions. The chemical constituents (-)-3-O-galloylcatechin and (-)-3-O-galloylepicatechin from *B. ciliata*, stigmasterol, quercetin, and avicularin from *M. pudica* and chebulagic acid, chebulic acid, corilagin, gallic acid, and ellagic acid from *P. emblica* might be responsible for antioxidant and enzyme inhibitory activity from ethyl acetate fraction. As a whole, our findings support indigenous practices of using *B. ciliata, M. pudica,* and *P. emblica* as therapeutic herbs and provide the basis for their effective use as a significant inhibitor of *α*-amylase and *α*-glucosidase for the treatment of diabetes.

## Figures and Tables

**Table 1:** Details of medicinal plants under study, their collection sites, parts used, and percentage yield.

Voucher specimen	Medicinal plants	Local name	Parts used in research	% yield (w/w)	Collection site	Altitude (m)	Geographical coordinates
BS-02	*B. ciliata*	Pakhanbhed	Stem	15.86	Shantipur, Gulmi, Nepal	1490 m	27°39′48.3″ N 83°28′52.3″ E
BS-04	*M. pudica*	Lajawati	Whole part	17.6	Shankar Nagar, Butwal, Nepal	158 m	28°11′24″ N 82°13′48″ E
BS-05	*P. emblica*	Amla	Fruit	39.5			

**Table 2:** Phytochemical identification of extracts.

		*B. ciliata*					*M. pudica*					*P. emblica*				
Phytochemical	Test performed	C	H	D	E	A	C	H	D	E	A	C	H	D	E	A
Alkaloids	Dragendorff's	+	+	−	+	+	+	−	−	+	+	+	+	+	+	+
Flavonoids	Alkaline reagent	+	+	+	+	+	+	+	+	+	+	+	+	−	+	+
Phenols	Ferric chloride	+	+	+	+	+	+	+	+	+	+	+	+	+	+	+
Steroids	Steroid test	−	−	−	+	+	−	−	−	+	+	−	−	−	+	+
Terpenoids	Salkowski	+	+	+	+	+	+	+	+	+	+	+	+	+	+	+
Tannins	Braemer's	+	+	−	+	+	+	+	−	+	+	+	+	−	+	+
Glycosides	Keller-Kiliani	+	+	+	+	+	+	−	+	+	+	+	+	+	+	−
Saponins	Foam	+	+	−	+	+	+	−	−	−	+	−	−	−	−	+
Carbohydrate	Molisch's	+	+	+	+	+	+	+	+	+	+	+	+	+	+	+
Anthraquinones	Anthraquinones	+	+	−	+	+	+	−	−	+	−	+	+	−	+	−

C: crude, H: hexane, D: dichloromethane, E: ethyl acetate, and A: aqueous.

**Table 3:** Total phenolic and total flavonoid contents of methanolic extracts and their fraction.

TPC (mg GAE/g)						TFC (mg QE/g)				
Medicinal plants	Crude	Hexane	DCM	EA	Aqueous	Crude	Hexane	DCM	EA	Aqueous
*B. ciliata*	155.83 ± 1.51	128.24 ± 1.22	87.86 ± 2.45	168.24 ± 1.17	172.58 ± 2.37	47.26 ± 1.21	26.25 ± 1.63	11.28 ± 0.10	28.49 ± 0.67	19.60 ± 3.10
*M. pudica*	131.78 ± 1.53	41.45 ± 2.6	66.81 ± 0.54	164.21 ± 1.81	152.01 ± 0.53	20.11 ± 0.75	11.77 ± 1.18	10.64 ± 0.32	64.89 ± 4.70	16.46 ± 2.42
*P. emblica*	171.73 ± 1.22	48.14 ± 2.57	74.78 ± 5.30	172.26 ± 3.61	154.62 ± 3.29	13.12 ± 0.29	18.07 ± 0.52	5.86 ± 0.55	48.04 ± 0.91	20.98 ± 3.36

DCM: dichloromethane; EA: ethyl acetate.

**Table 4:** Antioxidant activity of different fractions of selected plants.

Medicinal plants	DPPH radical scavenging (IC_50_ value, *μ*g/mL)				
	Crude	Hexane	DCM	EA	Aqueous
*B. ciliata*	52.60 ± 3.63	60.24 ± 2.19	108.20 ± 2.73	18.42 ± 1.29	16.99 ± 2.56
*M. pudica*	34.35 ± 5.11	81.28 ± 8.23	118.10 ± 0.76	21.39 ± 3.76	95.06 ± 3.03
*P. emblica*	22.36 ± 1.95	141.53 ± 10.73	88.85 ± 10.59	11.98 ± 0.36	22.34 ± 2.71
Quercetin (control)	2.86 ± 0.51				

**Table 5:** *α*-Amylase and *α*-glucosidase inhibition and their IC_50_ values of different fractions from medicinal plants.

Medicinal plants	IC_50_ value (*μ*g/mL)									
	*α*-Amylase					*α*-Glucosidase				
	Crude	Hexane	DCM	EA	Aqueous	Crude	Hexane	DCM	EA	Aqueous
*B. ciliata*	59.68 ± 0.69	50.84 ± 2.17	11.10 ± 0.33%^*∗*^	38.50 ± 1.32	74.26 ± 1.66	26.30 ± 0.56	40.74 ± 1.16	292.97 ± 0.55	3.41 ± 0.04	8.09 ± 0.28
*M. pudica*	114.83 ± 5.15	337.60 ± 10.33	29.79 ± 1.09%^*∗*^	110.90 ± 1.61	40.54 ± 0.63%^*∗*^	13.50 ± 0.56	14.17 ± 0.26	17.59 ± 0.43%^*∗*^	21.02 ± 0.78	16.62 ± 0.32
*P. emblica*	9.69 ± 1.28%^*∗*^	9.08 ± 0.94%^*∗*^	3.49 ± 0.86%^*∗*^	306.20 ± 18.5	8.22 ± 1.13%^*∗*^	80.62 ± 6.45	282.60 ± 4.98	14.79 ± 0.06%^*∗*^	11.48 ± 0.77	70.52 ± 3.65
Acarbose	3.13 ± 0.14 (*μ*g/mL)					2.06 ± 0.07 (mg/mL)				

^*∗*^Percentage inhibition at 500 *μ*g/mL.

**Table 6:** Correlation analysis of phenolics, flavonoids, antioxidants, and enzyme inhibitory effect.

	TPC	TFC	% DPPH scavenging	% *α*-amylase inhibition	% *α*-glucosidase inhibition
TPC	1				
TFC	0.553^*∗*^	1			
% DPPH scavenging	0.611^*∗*^	0.375	1		
% *α*-amylase inhibition	0.436	0.652^*∗*^^*∗*^	0.087	1	
% *α*-glucosidase inhibition	0.522^*∗*^	0.456	−0.012	0.637^*∗*^	1

^*∗*^Correlation is significant at the 0.05 level (2-tailed). ^*∗*^^*∗*^Correlation is significant at the 0.01 level (2-tailed).

**Table 7:** Principal component analysis for phytochemical content and pharmacological activities of different extracts of selected plants.

Principal component analysis (PCA)					
	F1	F2	F3	F4	F5
Eigenvalue	2.79	1.21	0.54	0.27	0.19
Variability (%)	55.81	24.23	10.87	5.36	3.75
Cumulative (%)	55.81	80.03	90.90	96.25	100.000

Factor loading	F1	F2	F3	F4	F5
TPC	0.831	0.335	−0.333	−0.043	0.290
TFC	0.834	0.003	0.451	−0.317	−0.015
DPPH	0.492	0.828	0.017	0.153	−0.220
*α*-Amylase	0.791	−0.421	0.245	0.366	0.058
*α*-Glucosidase	0.732	−0.485	−0.411	−0.089	−0.226

## Data Availability

The datasets used in this study are available upon reasonable request to the corresponding author.
